# 3D printing of composites of Martian regolith simulants and cyanobacterial biomass towards sustainable material production on Mars

**DOI:** 10.1038/s41526-025-00521-9

**Published:** 2025-08-29

**Authors:** Sophia Mannes Guesser De Oliveira, Kurosch Rezwan, Cyprien Verseux, Michael Maas

**Affiliations:** 1 Advanced Ceramics, University of Bremen, Bremen, Germany; 2 MAPEX, Centre of Materials and Processes, University of Bremen, Bremen, Germany; 3 Center of Applied Space Technology and Microgravity (ZARM), University of Bremen, Bremen, Germany

**Keywords:** Engineering, Materials science

## Abstract

The long-term goal of establishing a sustained human presence on Mars requires the capacity to produce essential consumables on-site. To this end, we develop strategies for processing inorganic oxidic powders and biomass into highly particle-filled composites using direct ink writing (DIW) 3D printing. Our approach relies on a simulant of a Martian regolith unit rich in hydrated clay minerals and food-grade spirulina, used as proxies for local regolith and cyanobacterial biomass, respectively. The composites are further reinforced through crosslinking with the plant-based molecule genipin. Detailed rheological analysis was performed for the 3D printing feedstocks, while the printed composites were characterized using thermal gravimetric analysis (TGA), surface area porosity analysis (BET), microscopy and mechanical tests. Dissolution tests demonstrated that genipin effectively crosslinks the cyanobacterial biomass. The outcome is a highly porous, lightweight material with adaptable, complex morphology, which has significant potential for use in the resource-constrained environments of long-duration Mars missions.

## Introduction

The goal of leading space agencies of crewed missions to Mars within the next decades appears realistic if one assumes that a sustained and intense political support will be provided^1^. Early missions could rely primarily on consumables hauled from the Earth. However, missions with a duration which is long enough to enable the extensive scientific exploration of the planet will require a higher reliance on materials produced on site, from resources naturally available there.

Martian regolith is the most abundant of these resources and could be used as the bulk component of structural materials. Its composition has been studied through Martian meteorites, landers, rovers, and orbiting spacecraft^2–7^. While its physical characteristics vary, notably due to differences in particle size and cohesion, its basaltic composition appears consistent across landing sites, suggesting a global homogenizing process^8^. Particle geometry was assessed by atomic force microscopy, showing irregular shapes with rounded edges^9^. Assessments of particle size distribution, though challenging due to instrument resolution limits, indicate that clay-sized particles (<4 μm) represent ca. 15–25 wt.% in soils and 75–85 wt.% of the <2 mm-materials in silt and sand^10^. More details on particle structure at specific sites can be found, for instance, in refs. ^11,12^.

In the design and selection of processes for joining regolith particles into stable ceramics, composites or other types of materials, considerations on energy costs and the availability of non-regolith components must weigh heavily. Sintering of regolith using solar lenses or lasers has been discussed and tested for lunar regolith and will result in mostly dense and hard ceramic materials^13^. Lighter and more flexible materials could be produced from combinations of regolith and organic polymeric materials.

A possible route for the in situ production of organic molecules is the cultivation of cyanobacteria. Previous work has shown that some cyanobacteria of the *Nostocaceae* family could be fed using Mars’s atmosphere as a source of carbon and nitrogen, regolith as a source of mineral nutrients, and water and sunlight to support photosynthesis; they could therefore be a relatively abundant source of organic materials in long-term Mars missions^14^. Other species of microalgae may be available as a byproduct of biological life-support processes: the single-celled green alga *Chlorella vulgaris*, for instance, is being considered for the production of oxygen and food^15,16^, and the cyanobacterium *Limnospira indica* (one among the species vernacularly referred to as “spirulina”) is expected to fulfill various bioregenerative functions within ESA’s MELiSSA closed-loop life support system^17^. Microalgal biomass contains an abundance of natural polymers (e.g., polysaccharides, proteins) of high molecular weight, polar and hydrogen-bonding functional groups, and potential sites for derivatization or cross-linking (e.g., amine, carboxyl, and hydroxyl groups). It could therefore serve as an important component of materials produced on Mars, for example, as an organic binder for regolith-based ceramics or composites.

Because of their versatility and ability to produce virtually any desired shape, 3D printing methods are expected to become essential to material production in extended space missions. Various additive manufacturing technologies have therefore been considered for shaping regolith. Among the most studied options are direct melting, sintering, and its use as an aggregate in concrete^18,19^. However, each among these approaches has severe limitations: they are energy intensive, extensively affected by environmental factors in the lunar or Martian environments (e.g., vacuum or very low atmospheric pressure, and wide-ranging temperature cycles), and/or can only generate simple geometries. Another option is the direct ink writing (DIW) of composites of regolith and organics^20,21^, which can be less energy-demanding and more flexible, and which can lead to lighter materials.

DIW, also known as robocasting or extrusion printing, is a 3D printing technique that extrudes a paste-like feedstock through a small nozzle while the printing head moves to deposit the extruded filament into the desired shape in a layer-by-layer pattern^22^. The feedstock material is typically a polymer gel or ceramic suspension which exhibits liquid-like behavior when exiting the nozzle but which retains its shape immediately after extrusion and after the deposition of successive layers^23^. Therefore, DIW relies on the extrusion of viscous suspensions with non-Newtonian rheological properties featuring a suitable yield point and thixotropic behavior^24,25^. Accordingly, the resolution and printing fidelity of DIW is governed by nozzle diameters, printing velocity, layer height and the rheological properties of the feedstock material. In previous work, we demonstrated the feasibility of DIW 3D printing various ceramic/hydrogel composites based on ceramic alumina particles and polysaccharides such as chitosan and alginate^26,27^. To reinforce the composite structures, the biocompatible and natural molecule genipin was used, which crosslinks amino groups (these groups are abundant in biologically sourced molecules like proteins or polyaminosaccharides like chitosane)^28^. Since biologically sourced binders are highly water soluble, covalent crosslinking is necessary to prevent humidity from causing the swelling or dissolution of the composites.

**Fig. 1:**
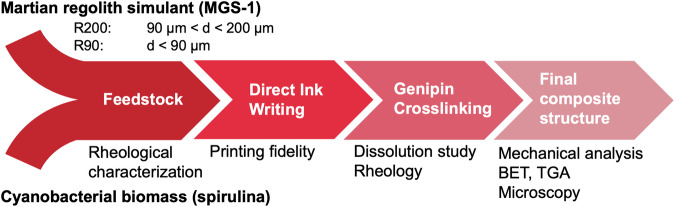
Flow chart of the experimental design.

Here, we demonstrate that Martian regolith-based composite materials can be produced using cyanobacterial biomass as a binder. We then optimize their composition for shaping into complex parts with DIW. Our experiments rely on a simulant of a Martian regolith unit rich in hydrated clay minerals and, as a proxy for cyanobacterial biomass produced on site, a lyophilized powder of the cyanobacterium *Arthrospira platensis* (commonly referred to as “spirulina”)^29^. The viscoelastic feedstocks for DIW are formulated in conjunction with rheological testing and the printed parts are characterized with various established methods including microscopy, thermogravimetry, BET and mechanical tests. The effect of covalent crosslinking with genipin on the stability against decomposition of the samples in water is assessed in dissolution experiments. Figure 1 provides an overview of the experimental work.

## Results and discussion

### General material characterization

Two basic types of composites were tested, based on two size fractions of the regolith simulant, along with pure components for comparison. The compositions we focused on were formulated after extensive testing and optimization, and only results for these final compositions (whose differences highlight the most important parameters) are shown (Table 1).

**Table 1:** Composition of the mixtures used as 3D printing feedstock in the present study

Sample Name	R200 (wt.%)	Spirulina (wt.%)	Genipin (wt.%)	Water (wt.%)
R200_0_S33_G0	–	33.33	**–**	66.67
R200_62_S0_G0	61.90	–	**–**	38.10
R200_44_S19_G0	42.86	19.05	**–**	38.10
**Sample Name**	**R90 (wt.%)**	**Spirulina (wt.%)**	**Genipin (wt.%)**	**Water (wt.%)**
R90_0_S43_G0	–	42.86	–	57.14
R90_0_S43_G008	–	42.85	0.0076	57.13
R90_0_S43_G034	–	42.84	0.0344	57.13
R90_0_S43_G084	–	42.83	0.084	57.10
R90_0_S43_G17	–	42.79	0.172	57.06
R90_7_S40_G0	6.67	40.00	–	
R90_7_S40_G007	6.67	39.99	0.0072	53.33
R90_7_S40_G032	6.67	39.99	0.032	53.32
R90_7_S40_G08	6.67	39.97	0.08	53.29
R90_7_S40_G16	6.67	39.94	0.16	53.24

**Fig. 2:**
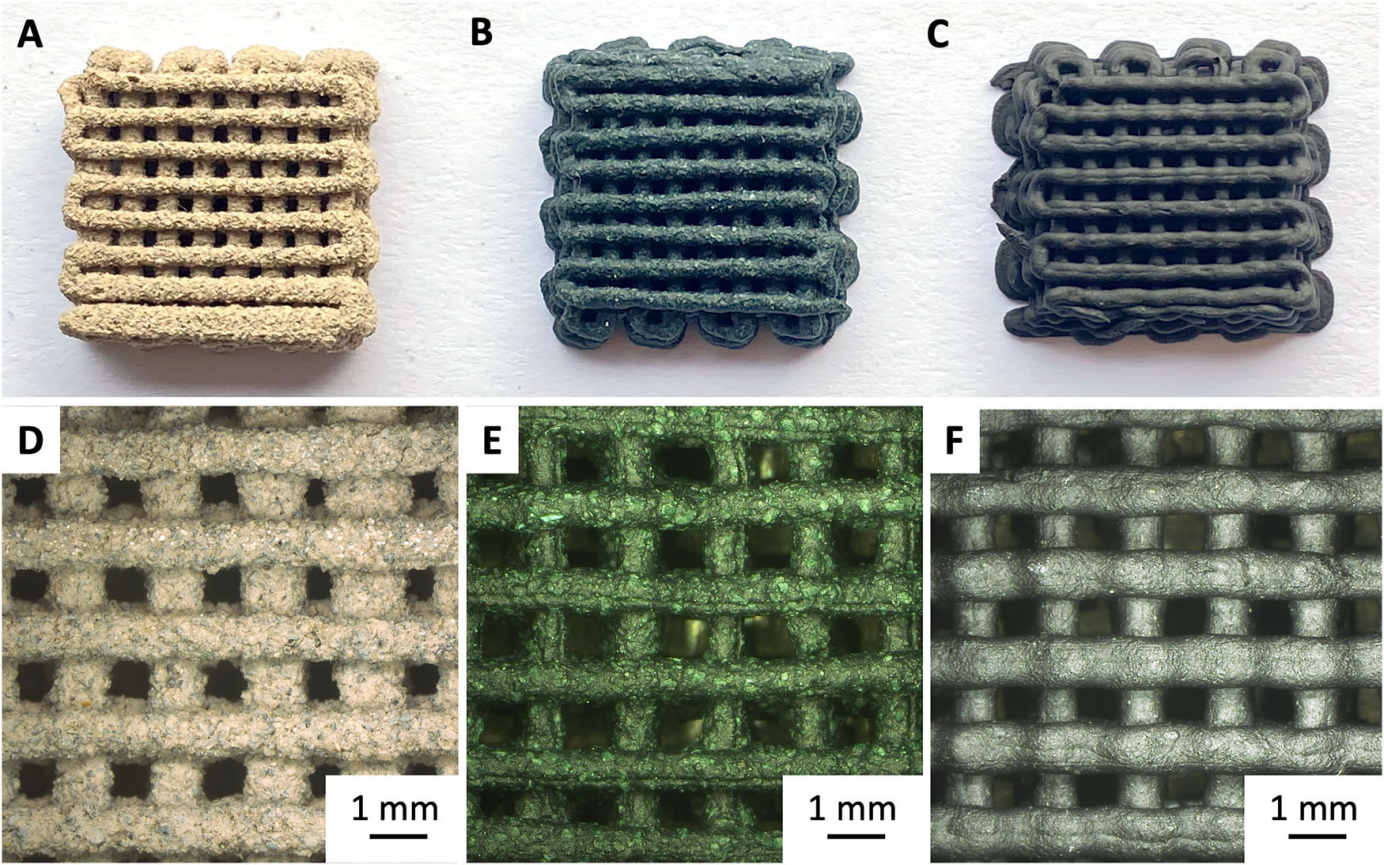
Images of printed structures. Photographs (**A**–**C**) and optical microscopy images (**D**, **E**) of printed samples R200_62_S0_G0 (**A**, **D**), R200_S19_G0 (**B**, **E**), and R90_7_S40_G0 (**C**, **F**).

The first composition includes regolith particles in the micrometer range with diameters between 200 µm and 90 µm (R200) as the main component and cyanobacterium biomass (30% of the dry weight) as a binder. Figure 2A, B shows representative parts printed using R200 with and without spirulina. The second type of feedstock is composed of micrometer and submicrometer sized regolith particles with a size fraction below 90 µm (R90) and a larger fraction of spirulina (six sevenths of the dry weight), which together form a hydrogel matrix. This matrix was reinforced by covalent crosslinking with various amounts of genipin. Parts printed using R90 have a slightly smoother appearance due to the smaller particle size and a darker green color due to the higher spirulina content (Fig. 2C).

Microscopy close-ups of representative printed lattice structures are shown in Fig. 2D–F. While the overall shape fidelity is good, some irregularities typically appear at the beginning or the end of the printing process. Slight fluctuations in printing pressure caused a variation in strut thickness of around 10%. The quality of the printed lattice structures can be semi-quantitatively assessed by using the printability test established by Ouyang et al.^30^, which is based on analyzing the regularity and rectangular shape of the voids between the lattice struts (Fig. 2D–F). In this test, a Pr value of 1 is ideal; a value higher indicates an elliptical elongation. Here, the average Pr value over all samples is 1.06 ± 0.097 and does not vary significantly between feedstocks.

**Fig. 3:**
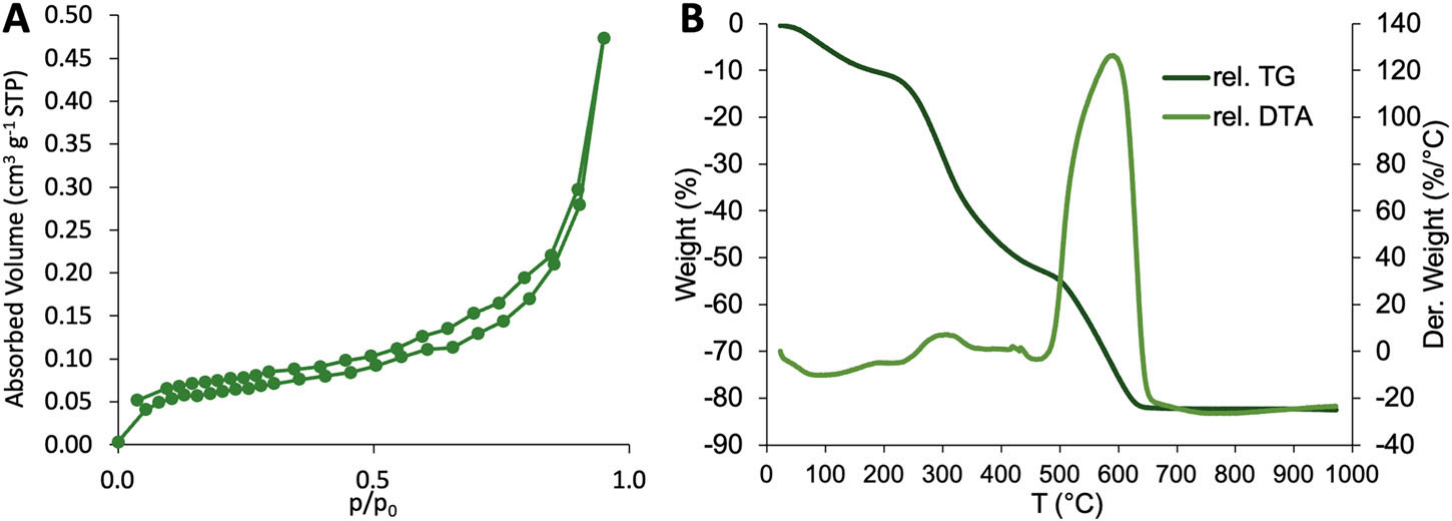
Material analysis data. **A** N_2_-adsorption/desorption isotherm and **B** TGA/DTA curve of sample R90_7_S40_G032.

Figure 3 shows results from further material analyses for a representative sample (R90_S40_G032): a BET nitrogen adsorption/desorption analysis and a thermogravimetric analysis. The former shows a low overall surface area of around 0.2 m^2^ g^-1^, even though the type 4 isotherm hints at the presence of mesoporosity (Fig. 3A). In this, the sample shows typical properties of a dried hydrogel without accessible pores. The latter (the thermogravimetric analysis, Fig. 3B) sheds light on the temperature-dependent decomposition of the samples. Weight variations over temperatures ranging from room temperature to 100 °C reveal that this specific sample, even after drying for 3 h at 80 °C, still contains 10 wt.% water, which can be explained by the hygroscopic behavior of spirulina. Between 100 and 500 °C, the sample loses approximately 50% of its weight, which can be attributed to the decomposition and evaporation of the spirulina. Between 500 and 700 °C, the sample undergoes an exothermic decomposition at which the remainder of the spirulina content is removed. At higher temperatures, only the inorganic and temperature-resistant regolith remains and the sample weight stays unchanged until the end of the measurement at 1000 °C.

### Rheological characterization of feedstocks for 3 d printing

**Fig. 4:**
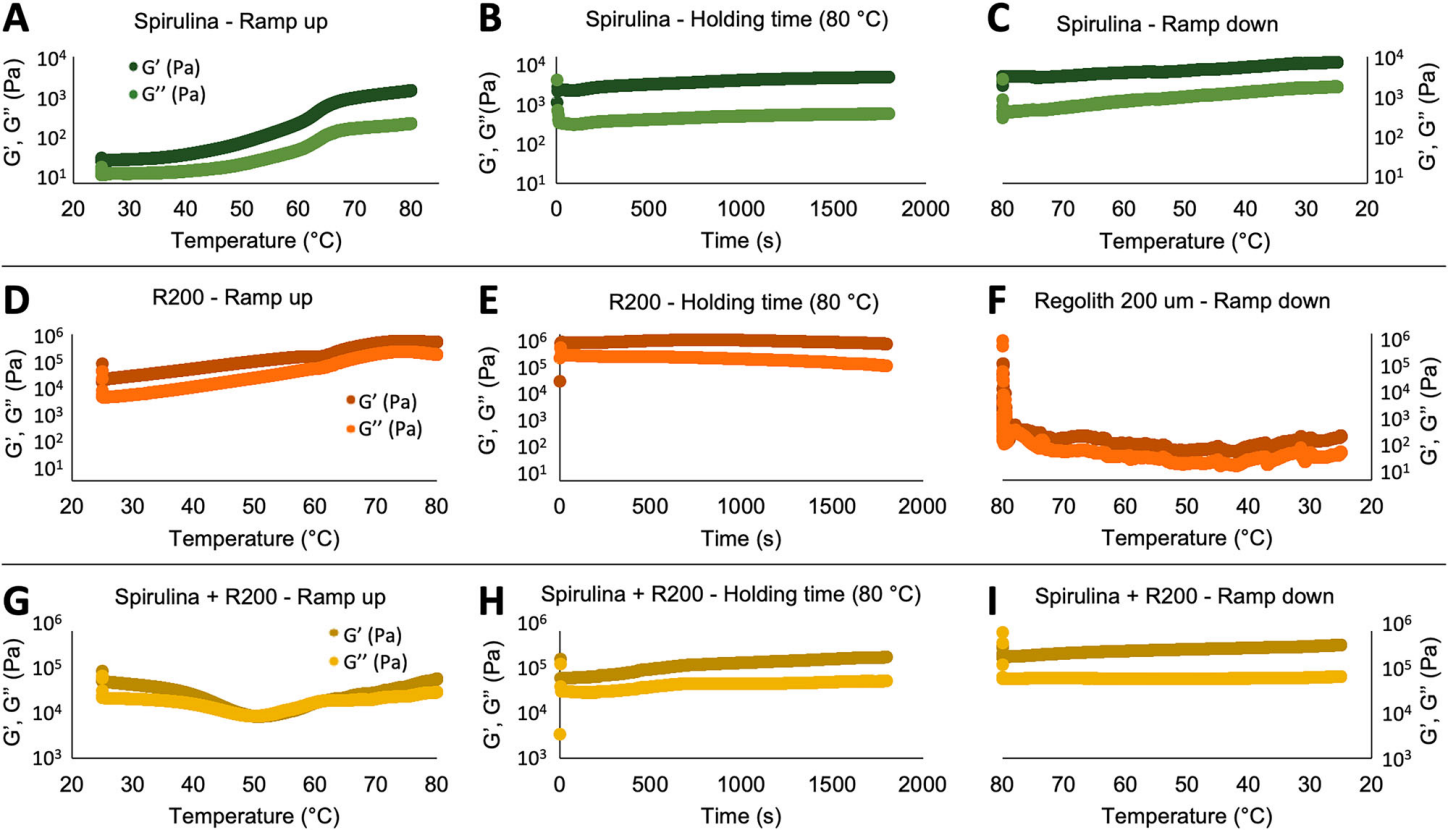
Temperature-dependent viscoelastic moduli of spirulina and R200 suspensions. **A**–**C** Spirulina in water (R200_0_S33_G0); **D**–**F** regolith in water (R200_62_S0_G0); **G**–**I** both materials combined (R200_44_S19_G0). **A**, **D**, **G** Temperature ramp from 25 to 80 °C; **B**, **E**, **H** 30 min holding time at 80 °C; **C**, **F**, **I** temperature ramp from 80 to 25 °C.

Non-Newtonian rheology analyses are critical to the development of DIW feedstocks. The viscosity of bio-based hydrogels often strongly increases at elevated temperatures due to the denaturation, flocculation and/or gelation of proteins and polysaccharides. Therefore, the temperature-dependent rheological behavior of spirulina and R200 suspensions was tested over a temperature range of 25–80 °C using oscillatory rheological measurements (Fig. 4A). These tests were carried out at an oscillatory strain at 1% close to the linear viscoelastic threshold and an oscillatory frequency of 1 Hz, and they provide the viscoelastic moduli G’ (the storage modulus, describing the elastic properties of the material) and G” (the loss modulus, describing the viscous properties of the material) as a function of temperature.

Figure 4 shows representative results from these measurements. Each row with three graphs represents a single measurement run, where the sample is first heated up from 25 to 80 °C, held for 30 min at 80 °C and finally cooled down from 80 °C to 25 °C. Spirulina in water shows a marked increase in elastic and viscous moduli with increasing temperature up to around 65 °C. Afterwards, the moduli remain constant, even after cooling. This behavior is rather typical and is also encountered, for instance, with starch-based suspensions during food processing. Suspensions of pure R200 only show minor changes in the moduli of the paste-like fluid, which are mostly caused by drying. Drying also causes the sharp drop in the final stage of the experiment, during which cracks parallel to the shearing plane form in the dried sample. Mixtures of R200 and about 20 wt.% spirulina show similar rheological properties to suspensions with just R200, but are less affected by drying, which highlights the capability of spirulina to retain water. Efforts aimed at improving the resource-efficiency of the proposed production process could be directed toward minimizing such water retention, and more broadly at recovering solvent water, as water extraction on Mars will be resource-intensive (see for instance Table 6-11 in the Addendum to the Mars Design Reference Architecture 5.0^31^).

**Fig. 5:**
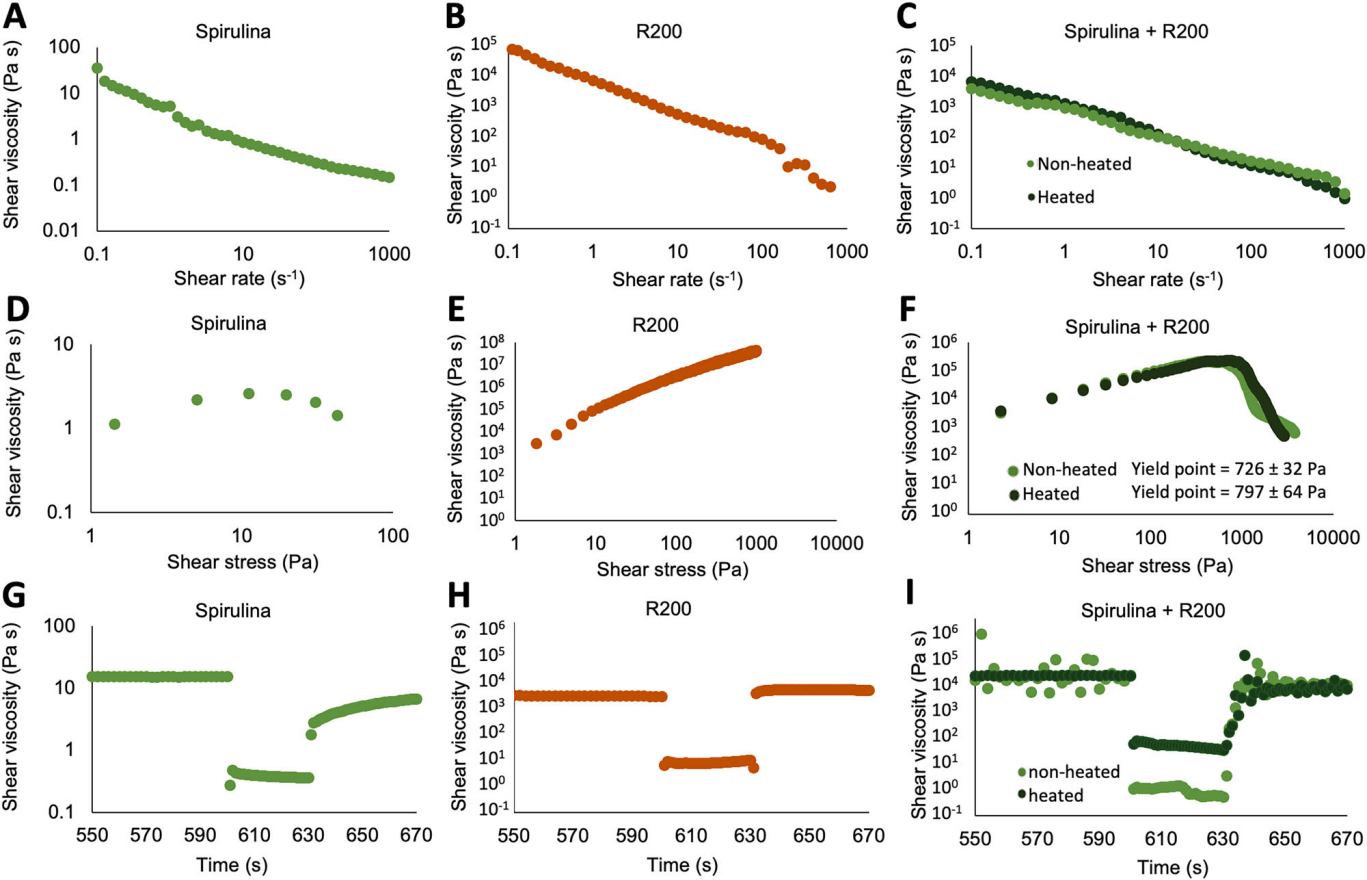
Shear rate test, shear stress test and thixotropy analysis of spirulina and R200 suspensions. Shear rate-dependent viscosity (**A**–**C**), shear stress-dependent viscosity (**D**–**F**) and time-dependent shear viscosity (**G**–**I**) of (**A**, **D**, **G**) spirulina in water (R200_0_S33_G0); (**B**, **E**, **H**) regolith in water (R200_62_S0_G0); and (**C**, **F**, **I**) both materials combined (R200_44_S19_G0).

The results of the shear rate tests (Fig. 5A–C) show shear thinning behavior for all tested materials, characterized by a decrease in the viscosity of the material with an increase in the shear rate. This behavior is favorable for DIW because an increased shear rate during extrusion of the feedstock facilitates the flow through the printing nozzle. Furthermore, although the moduli of heated spirulina are significantly higher than these of unheated spirulina, heating then cooling down the mixture of R200 and spirulina does not affect its shear rate properties.

A well-defined yield point with a shear stress range of 500–1000 Pa is also critical for the specific setup used in these experiments. It must be low enough to allow the feedstock to flow through the nozzle at reasonable hydraulic pressure (around 180 kPa for this printing setup) and high enough for the printed layers to withstand the pressure of subsequent layers without deformation or flow. The results of the shear stress tests (Fig. 5D–F) show that neither the spirulina suspension nor R200 at high solid loading have a well-defined yield point. Conversely, the mixture of both materials shows the desired results, with yield points of 726 ± 32 Pa for the non-heated and 797 ± 64 Pa for the heated feedstock R200_0_S33_G0, which can be determined from the shear stress at the steep decline in shear viscosity which indicates flow initiation. Again, in the mixture, prior heating of the spirulina does not significantly change the rheological characteristics of the feedstock.

Results from the thixotropy test of the different materials are represented in Fig. 5G–I. In this test, the feedstocks are first subjected to a low shear rate regime at 0.1 s^-1^ for 600 s to record the viscosity with little agitation. This simulates the state of the feedstock prior to passing through the printer’s extrusion nozzle. Subsequently, the shear rate increases rapidly to 100 s^–1^ and is held for 30 s, which corresponds to the higher shear rates encountered during extrusion. Finally, the shear rate is reduced again to the initial test conditions to simulate the material at rest. In DIW, it is critical that the feedstock quickly recovers its initial viscosity and yield point after the high-shear rate regime, ideally before the next layer is printed on top after several seconds. While spirulina shows a slow recovery time, R200 and the combination of both materials show a very fast recovery, below 5 s. Heating of the spirulina seems to slightly reduce the drop in viscosity under high shear rate, which would have little to no effect on the printing process.

**Fig. 6:**
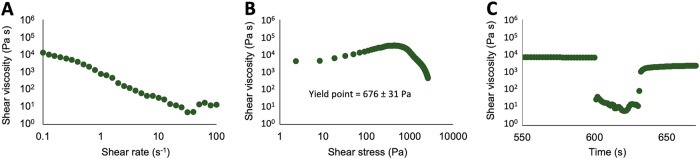
Shear rate test, shear stress test and thixotropy analysis of feedstocks on the basis of R90. Shear viscosity of feedstocks R90_7_S40_G0 as a function of shear rate (**A**); shear stress (**B**); and time (**C**).

Feedstocks composed of R90 and spirulina were subjected to the same shear rate, shear stress and thixotropy tests as discussed above and the results are shown in Fig. 6. Although the regolith particle concentration in these feedstocks was significantly less than with R200 (see Table 1), viscosity, shear thinning behavior, yield point of 676 ± 31 Pa and thixotropy are very similar. This can be attributed to stronger interactions between the particles, which have a higher surface-to-volume ratio. Accordingly, both types of compositions can be conferred rheological properties which are optimal for the DIW process.

### Influence of genipin on composite stability

**Fig. 7:**
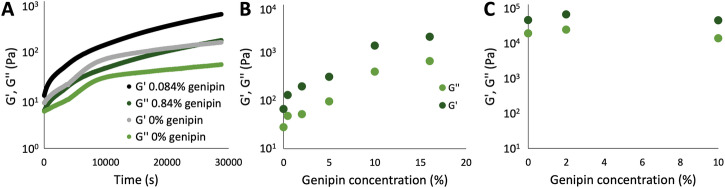
Influence of genipin crosslinking on the viscoelastic moduli of the feedstocks. **A** Viscoelatic moduli over time of spirulina in water, with or without genipin (R90_0_S43_G0 and R90_0_S43_G08); **B** viscoelastic moduli of spirulina in water as a function of genipin concentration (R90_0_S43_G0 to R90_0_S43_G16) after 5.56 h of crosslinking; **C** viscoelastic moduli after 5.56 h of a feedstock containing R90 and spirulina, as a function of genipin concentration (R90_7_S40_G0 to S90_7_S40_G16).

Finally, the concentration-dependent influence of genipin crosslinking on G’ and G” was analyzed with oscillatory rheology. To this end, 8 h time tests with fixed oscillatory strain and frequency were carried out (see Fig. 7A for an example). In Fig. 7B, C, G’ and G” after 20000 s (5 h and 33 min) of crosslinking at 25 °C are plotted as a function of genipin concentration in the feedstocks. The moduli strongly increase when genipin is added to pure spirulina solutions up to around 10% of genipin, in relation to the mass of spirulina (Fig. 7B). As with the results reported above, the effect is much less pronounced in the presence of R90 in the feedstock, which strongly dominates the rheological properties (Fig. 7C). The amplitude tests show typical behavior with a linear viscoelastic range until around 1% strain amplitude, after which the moduli sharply decrease (data not shown).

**Fig. 8:**
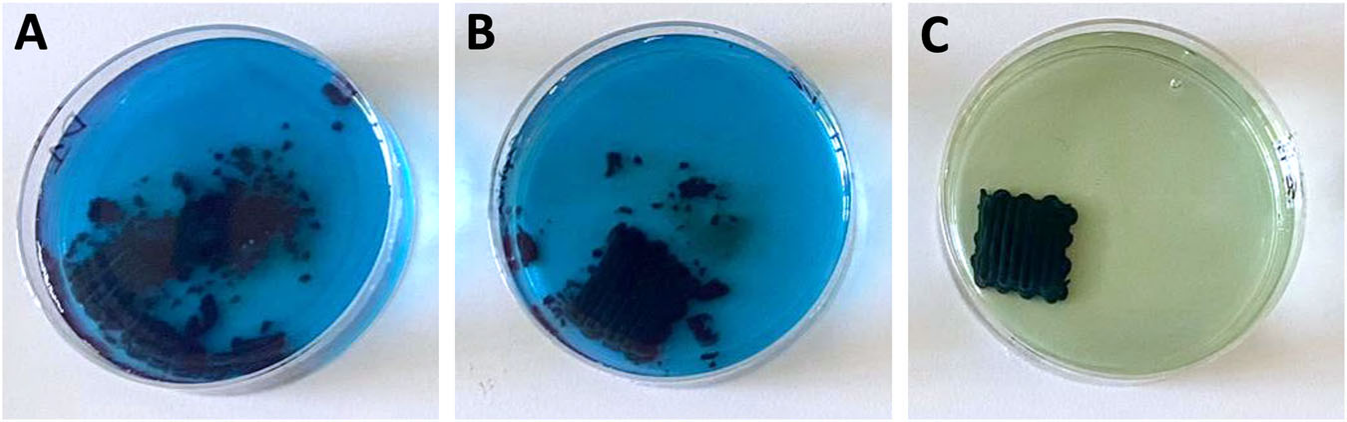
Printed samples with increasing genipin concentration after 24 h in water. **A** R90_7_S40_G0; **B** R90_7_S40_G007; and **C** R90_7_S40_G16.

Although the addition of genipin does not have a strong impact on the rheology of the composites, crosslinking of spirulina significantly delays dissolution in water. This is qualitatively shown in Fig. 8 for composites with feedstocks R90_7_S40_G0, R90_7_S40_G072 and R90_7_S40_G16, which were submerged in water for 24 h under gentle shaking. Without genipin crosslinking, the samples rapidly disintegrate and partially dissolve (Fig. 8A). The addition of genipin at only 0.0072 wt.% already prevents most of the sample dissolution within 24 h. A genipin concentration of 0.16 wt.% clearly stabilizes the sample against dissolution and enables shape retention throughout three days of immersion in water (not shown).

**Fig. 9:**
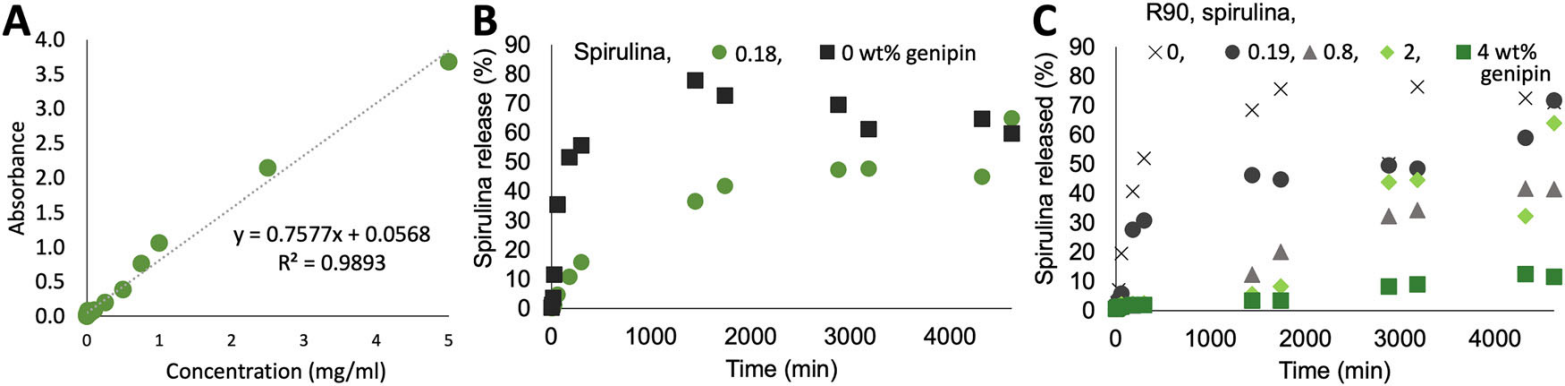
Dissolution analysis of feedstocks with varying genipin concentration. **A** Calibration curve for determining the concentration of spirulina in water; **B** spirulina release over time (as a metric for dissolution dynamics) for samples R90_0_S43_G0 and R90_0_S43_G008; and **C** spirulina release over time from samples comprising spirulina, R90 and various concentrations of genipin (R90_7_S40_G0 to R90_7_S40_G16).

**Fig. 10:**
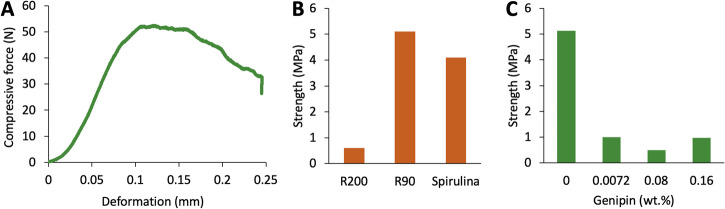
Compression tests of 3 d printed lattice structures. **A** Representative stess/strain graph of composition R90_7_S40_G0, **B** effective strength of the composites R200_44_S19_G0, R90_7_S40_G0 and R90_0_S43_G0 (pure spirulina) and **C** the effect on genipin on the strength of composites R90_7_S40_GX with X = genipin concentration.

In order to quantify the spirulina content in the medium surrounding the immersed materials over time, and thus the dynamics of dissolution of spirulina, a calibration curve was generated based on the Beer-Lambert law (Fig. 9A). Figure 9B shows the spirulina release from a sample printed from pure spirulina, as well as from pure spirulina cross-linked with a low amount of genipin. Only 50% of the spirulina is released after 12 h from the cross-linked sample, while the sample without genipin completely disintegrates after 30 min. The same trend can be observed for composites of R90 and spirulina (Fig. 10C). The spirulina release decreases with genipin concentration up to 0.16 wt.%, at which spirulina release is prevented almost completely.

### Mechanical characterization of the composites

Compression tests of the dried composites show that all composites are characterized by an effective compressive strength of a few MPa, which is enough to safely handle the materials for further processing (Fig. 10). While the R200 composites are quite brittle and show low strain tolerance, the stress/strain curve of the R90 composites is similar to that of deformable polymer foams with a maximum strength at around 5% strain and progressive collapse of the structure at higher deformations (Fig. 10A). The composite with R90 and spirulina (R90_0_S43_G0) shows the highest strength of about 5 MPa, which is surprisingly similar to the pure spirulina structure (Fig. 10B). The composite with larger particle size and less spirulina is quite brittle, with a strength of around 0.6 MPa (R200_44_S19_G0). Crosslinking spirulina with genipin further reduces the strength of the composites to about 1 MPa, probably due to an increased brittleness of the cross-linked structures (Fig. 10C). Conversely, as discussed above, the rheological experiments demonstrate much higher viscosity and moduli for the regolith composites with spirulina than for pure spirulina and also showed a reinforcing effect of genipin crosslinking with pure spirulina. Accordingly, the properties of the wet composites that are optimized for DIW 3 d printing do not fully translate to the dried structures. Further processing will be necessary to strengthen the composites for load-bearing applications.

In summary, this study demonstrates that materials with promising properties can be produced from Martian regolith and cyanobacterial biomass. By optimizing the composition of the resulting composites, we successfully tailored their rheological properties for DIW. The resulting feedstocks are suited for creating complex shapes while maintaining structural integrity after extrusion, with compressive strength of the dried structures in the order of a few MPa. The cyanobacterial biomass proved to be an effective organic binder, and the crosslinking of its amine groups with genipin significantly enhanced the stability and durability of the printed composites.

Our results are only a first step. Extensive work will be required, for instance, to determine and enhance the durability of the printed objects in the targeted environments, be they inside shielded infrastructure or outdoors. Further efforts should focus on quantifying and enhancing the resource-efficiency of the process. The use of water as a solvent, for instance, will be costly, and future work could aim at reducing its use and recovering a high fraction of it. The consumption of cyanobacterium biomass, if produced for that sole purpose, may be costly as well^32^ – though such biomass (as well as that of other microorganisms) may be expected to be a byproduct of various microbial processes on Mars^33^. Another important aspect to study will be the influence of the reduced Martian gravity on the printing process. In this respect, the DIW process could benefit from the decreased weight of the stacked layers, but slight variations in dynamics and normal forces could offset the balance set by the feedstocks’ complex rheological properties^19^. Overall, our findings underscore the feasibility of leveraging Martian regolith and biologically sourced binders for material production in long-duration missions. The suitability of the resulting materials for 3D printing by DIW opens up a wide range of possibilities for constructing varied and complex materials directly on Mars, contributing to the overall success of efforts towards the exploration of the red planet.

## Methods

### Materials

The Mars Global Hydrated Clay (MGS-1C) Martian Dust Simulant was obtained from Space Resource Technologies (FL, USA) and sieved to remove larger particles that would block the printer and to homogenize the particle sizes. Two particle size fractions were prepared: 90–200 µm (R200) that presents a fraction only based on particles in the micrometer range and below 90 µm (R90) which also contains very small particles with high surface area to volume ratio. Organic powder of *Arthrospira platensis* (hereafter referred to as spirulina) was purchased from borchers fine food GmbH & Co. KG (Oyten, Germany) and used as a proxy for cyanobacterial biomass produced on Mars.

### Slurry preparation

All feedstocks were prepared by mixing 4 mL water, and regolith (R90 or R200), spirulina and genipin at the concentrations listed in Table 1, with the aid of a mechanical disperser (Dispermat® model d-51580; VMA‒GETZMANN, Reichshof, Germany) for 5 min at 1000 rpm. The resulting feedstocks were either directly processed with a 3D printer (see below) or crosslinked as follows. A 4% (w/v) stock solution of genipin was prepared by dissolving 0.2 g of genipin in 1 mL ethanol using ultrasonication for 15 min, followed by the addition of 4 mL of water. The solution was incorporated into the feedstocks at different concentrations. Table 1 lists the composition of the resulting mixtures, which were used as 3D printing feedstock (see below). The wt.% values are calculated from the mixing ratios on the basis of 4 mL water with the addition of 2 g (R200 formulations) or 3 g (R90 formulations) of spirulina and 0.5 g R90, 4.5 g R200 or 6.5 g R200, respectively. For reference, with densities of 2.89 g/cm^3^ (R200) and 2.66 g/cm^3^ (R90), the volume fraction of the regolith is 36% (w/v) in the composition R200_62_S0_G0 and about 2% (w/v) in R90_7_S40_G0. Because of its partial solubility in water, the volume fraction of spirulina in the mixture cannot be accurately provided. With a molecular weight of 226.226 g mol^-1^, the molarity of genipin at 0.0072 wt.% is 0.14 mM (in relation to the water content) in R90_7_S40_G007 and 3.18 mM in R90_7_S40_G16.

### 3D printing

The feedstocks were printed into lattice cubes (1 × 1 × 0.5 cm) using the printer Inkredible (Cellink, Gothenburg, Sweden) in a 6-well plate, using a conical precision tip nozzle (⌀ 940 μm) with an extrusion air pressure of 180 ± 5 kPa, a printing speed of 10 mm/s, and a printing temperature of 25 °C. The printing commands were generated in the 3D printing programming language G-code using the HeartWare 2.4.1 software (Cellink), with a 40% infill density and 0.85 mm layer height. After printing, the samples were dried in a drying cabinet (Heraeus Electro-Nite GmbH & Co KG, Hagen, Germany) for 3 h at 80 °C.

### Rheological tests

The rheological properties of feedstocks with printable viscosity were determined using a Kinexus Pro rheometer (Malvern Panalytical, Malvern, UK). A temperature test was first performed to assess the influence of increasing temperature on the viscoelastic moduli of spirulina and the composites. The feedstocks were heated from 20 to 80 °C, which was followed by a 30 min isothermal step and a cooling step to the initial temperature during which the elastic modulus (G’) and the viscous modulus (G”) of the composite materials were recorded at a constant strain of 1% and a constant oscillation frequency of 1 Hz. A thixotropy test was performed to determine the feedstocks’ viscosity recovery time after a period of high shear rate, as occurs in extrusion during 3D printing. To this end, the samples were first sheared at a shear rate of 0.1 s^–1^ for 600 s, followed by a shear rate of 100 s^–1^ for 30 s and finally again 0.1 s^–1^ for 600 s. A stepped shear rate test was performed with 1 s of equilibration time between each change in shear rate (10 steps per decade). Continuous shear stress sweeps were used to record the shear stress as a function of shear rate to determine the yield points of the samples. Samples containing genipin were further subjected to a single frequency oscillatory test for 8 h to evaluate the relationship between genipin concentration and the changes in viscoelastic properties of the composite materials (constant strain of 0.1% and constant oscillation frequency of 1 Hz). The same samples were then subjected to amplitude sweep tests at a constant oscillation frequency of 1 Hz.

### Optical microscopy and printability characterization

Optical microscopy images were obtained using a VHX-5000 digital light microscope (Keyence Deutschland GmbH, Neu-Isenburg, Germany) to verify the quality and fidelity of the 3D printing. Images with ×20, ×30, and ×50 resolution were taken for three printed objects per feedstock composition.

The printability of the feedstocks—namely, their capability of being shaped into 3D structures with good fidelity and integrity—was evaluated by means of image analysis. Ideally, printed constructs should display a clear morphology with smooth surfaces, constant diameters after printing, and the ability to stack with other filaments without merging. Therefore, for regular lattice grid structures, square-shaped holes should in principle be formed in the interstitial spaces between interconnected filaments in the fabricated constructs. Ouyang et al. proposed an approach to define bioink printability (Pr) based on the analysis of the hole shape using Eq. 1^30^:

1 $$\Pr =\frac{{L}^{2}}{16A}$$

with the hole perimeter *L* and the hole area *A*. Ideally, the Pr value should be 1, which is the case when the interconnected channels of the constructs form a square shape. To determine the Pr values of the printed samples, *L* and *A* were determined from optical microscopy images using the software Fiji (ImageJ2, version 2.14.0/1.54 f).

### Mechanical testing

Compression tests were performed with a universal testing machine (Zwick/Roell Z005). The air-dried samples were prepared by carefully grinding the top and bottom sides and a half sphere was positioned on the samples to assure that the force was uniformly applied to the whole lattice structure. A preload of 0.1 N was applied, after which the sample was compressed with a speed of 0.5 mm/min. The effective strength of the composites was determined by dividing the maximum force before breaking by the rectangular area of the specific sample. At least two samples were tested per sample composition.

### Dissolution testing

In order to test the stability of the composites in water, the printed samples were placed in a container with 15 ml of water and agitated with a mechanical shaker (GFL platform shaker 3015; GFL Gesellschaft für Labortechnik mbH, Burgwedel, Germany). Aliquots of 300 µl of the aqueous phase surrounding the sample were collected after 1 min, 5 min, 15 min, 30 min, 1 h, 3 h, 5 h, 24 h, 29 h, 48 h, 53 h, 58 h, 72 h and 77 h, and 300 µl of water was added after each collection step. The optical density of the collected liquid was measured at a wavelength of 681 nm using a Thermo Scientific Multiskan GO UV-visible spectrophotometer (Thermo Fisher Scientific, Waltham, MA, USA). To determine spirulina concentrations, a calibration curve was made beforehand, based on the Beer-Lambert law and using known concentrations of spirulina. For each type of feedstock (Table 1), three printed samples were analyzed.

### Thermogravimetric analysis (TGA)

A thermogravimetric analysis was performed on the Simultaneous Thermal Analyzer STA 503 (BÄHR-Thermoanalyse GmbH; now part of TA Instruments, Hüllhorst, Germany). The samples were heated from 25 to 1000 °C at a rate of 5 °C min^–1^, under an air flow of 20 ml/min.

### Surface area and porosity analysis (BET)

Nitrogen adsorption isotherms were performed with a Belsorp-Mini instrument (Bel Japan Inc.; now MicrotracBEL, Osaka, Japan) at −196 °C, after drying samples for 3 h at 100 °C under low pressure (<2 mbar).

## Data Availability

The data supporting the findings of this study are available from the corresponding author upon reasonable request.

## References

[1] ISECG. The Global Exploration Roadmap, 3rd edition.

[2] Clark, B. C. et al. Chemical composition of Martian fines. *J. Geophys. Res. Solid Earth* **87**, 10059–10067 (1982).

[3] Gellert, R. et al. Alpha Particle X-Ray Spectrometer (APXS): Results from Gusev crater and calibration report. *J. Geophys. Res. Planets* **111**, E02S05 (2006).

[4] Ming, D. W. et al. Geochemical properties of rocks and soils in Gusev Crater, Mars: Results of the Alpha Particle X-Ray Spectrometer from Cumberland Ridge to Home Plate. *J. Geophys. Res. Planets* **113**, E12S39 (2008).

[5] Vaniman, D. T. et al. Mineralogy of a Mudstone at Yellowknife Bay, Gale Crater. *Mars. Sci.* **343**, 1243480 (2014). 10.1126/science.1243480 24324271

[6] Siebach, K. L. et al. Sorting out compositional trends in sedimentary rocks of the Bradbury group (Aeolis Palus), Gale crater, Mars. *J. Geophys. Res. Planets* **122**, 295–328 (2017).

[7] Rampe, E. B. et al. Mineralogy and geochemistry of sedimentary rocks and eolian sediments in Gale crater, Mars: A review after six Earth years of exploration with Curiosity. *Geochemistry* **80**, 125605 (2020).

[8] *The Martian Surface: Composition, Mineralogy and Physical Properties*. (Cambridge University Press, Cambridge, 2008). 10.1017/CBO9780511536076.

[9] Pike, W. T. et al. Quantification of the dry history of the Martian soil inferred from in situ microscopy. *Geophys. Res. Lett.* **38**, L24201 (2011).

[10] Ming, D. W. & Morris, R. V. Chemical, Mineralogical, and Physical Properties of Martian Dust and Soil. in (Houston, TX, 2017).

[11] Ehlmann, B. L. et al. Chemistry, mineralogy, and grain properties at Namib and High dunes, Bagnold dune field, Gale crater, Mars: A synthesis of Curiosity rover observations. *J. Geophys. Res. Planets* **122**, 2510–2543 (2017). 29497589 10.1002/2017JE005267 PMC5815393

[12] Rivera-Hernández, F. et al. Using ChemCam LIBS data to constrain grain size in rocks on Mars: Proof of concept and application to rocks at Yellowknife Bay and Pahrump Hills, Gale crater. *Icarus* **321**, 82–98 (2019).

[13] Farries, K. W., Visintin, P., Smith, S. T. & van Eyk, P. Sintered or melted regolith for lunar construction: state-of-the-art review and future research directions. *Constr. Build. Mater.* **296**, 123627 (2021).

[14] Verseux, C. et al. Sustainable life support on Mars – the potential roles of cyanobacteria. *Int. J. Astrobiol.* **15**, 65–92 (2016).

[15] Detrell, G. Chlorella vulgaris photobioreactor for oxygen and food production on a moon base-potential and challenges. *Front. Astron. Space Sci* **8**, 700579 (2021).

[16] Casula, M. et al. Cultivation and nutritional characteristics of *Chlorella vulgaris* cultivated using Martian regolith and synthetic urine. *Life Sci. Space Res.* **42**, 108–116 (2024). 10.1016/j.lssr.2024.06.003 39067982

[17] Poughon, L., Creuly, C., Godia, F., Leys, N. & Dussap, C.-G. Photobioreactor limnospira indica growth model: Application from the MELiSSA plant pilot scale to ISS flight experiment. *Front. Astron. Space Sci* **8**, 700277 (2021).

[18] Snehal, K., Sinha, P. & Chaunsali, P. Development of waterless extra-terrestrial concrete using Martian regolith. *Adv. Space Res.* **73**, 933–944 (2024).

[19] Wang, Y. et al. In-situ utilization of regolith resource and future exploration of additive manufacturing for lunar/martian habitats: A review. *Appl. Clay Sci.* **229**, 106673 (2022).

[20] Taylor, S. L. et al. Sintering of micro-trusses created by extrusion-3D-printing of lunar regolith inks. *Acta Astronaut.* **143**, 1–8 (2018).

[21] Jakus, A. E., Koube, K. D., Geisendorfer, N. R. & Shah, R. N. Robust and Elastic Lunar and Martian Structures from 3D-Printed Regolith Inks. *Sci. Rep.* **7**, 44931 (2017). 28317904 10.1038/srep44931 PMC5357966

[22] Direct Ink Writing: A 3D Printing Technology for Diverse Materials - Saadi - 2022 - Advanced Materials - Wiley Online Library. https://onlinelibrary.wiley.com/doi/full/10.1002/adma.202108855. 10.1002/adma.202108855 35246886

[23] Decante, G. et al. Engineering bioinks for 3D bioprinting. *Biofabrication* **13**, 032001 (2021). 10.1088/1758-5090/abec2c 33662949

[24] Agrawal, R. & García-Tuñón, E. Interplay between yielding, ‘recovery’, and strength of yield stress fluids for direct ink writing: new insights from oscillatory rheology. *Soft Matter* **20**, 7429–7447 (2024). 39258474 10.1039/d4sm00758a PMC11388702

[25] Rau, D. A., Williams, C. B. & Bortner, M. J. Rheology and printability: A survey of critical relationships for direct ink write materials design. *Prog. Mater. Sci.* **140**, 101188 (2023).

[26] Condi et al. 3D bioprinting of hydrogel/ceramic composites with hierarchical porosity. *J. Mater. Sci.* **57**, 3662–3677 (2022).

[27] Condi Mainardi, J., Rezwan, K. & Maas, M. Embedding live bacteria in porous hydrogel/ceramic nanocomposites for bioprocessing applications. *Bioprocess Biosyst. Eng.* **42**, 1215–1224 (2019). 30953175 10.1007/s00449-019-02119-4

[28] Condi Mainardi, J., Rezwan, K. & Maas, M. Genipin-crosslinked chitosan/alginate/alumina nanocomposite gels for 3D bioprinting. *Bioprocess Biosyst. Eng.* **45**, 171–185 (2022). 34664115 10.1007/s00449-021-02650-3 PMC8732963

[29] Soni, R. A., Sudhakar, K. & Rana, R. S. *Spirulina* – From growth to nutritional product: A review. *Trends Food Sci. Technol.* **69**, 157–171 (2017).

[30] Ouyang, L., Yao, R., Zhao, Y. & Sun, W. Effect of bioink properties on printability and cell viability for 3D bioplotting of embryonic stem cells. *Biofabrication* **8**, 035020 (2016). 27634915 10.1088/1758-5090/8/3/035020

[31] *Human Exploration of Mars Design Reference Architecture 5.0 – Addendum*. (NASA, 2009).

[32] Ramalho, T. P. et al. Resource-efficiency of cyanobacterium production on Mars: Assessment and paths forward. *Algal Res.* **84**, 103801 (2024).

[33] Nangle, S. N. et al. The case for biotech on Mars. *Nat. Biotechnol.* **38**, 401–407 (2020). 32265561 10.1038/s41587-020-0485-4

